# Iritis

**Published:** 1890-02

**Authors:** T. Hilliard Wood

**Affiliations:** Nashville, Tenn.


### IRITIS.*

BY T. HILLIARD WOOD, M. D., NASHVILLE, TENN.

Inflammation may begin in, and remain confined to, the tissue of the iris, constituting iritis. Or it may extend from the iris by continuity of tissue to other parts, as to the ciliary bodies, giving rise to irido-cyclitis; or to the choroid, causing irido-choroiditis. In these cases the iritis is primary, the cyclitis or choroiditis secondary.

*Read before Nashville Academy of Medicine and Surgery, December 12, 1889.

Inflammations originally in the cornea, ciliary bodies or choroid, may extend to the iris, when the iritis is secondary.

The first changes observed in the iris are due to congestion of its vessels, this congestion being most intense in the greater and smaller circles of the iritis.

This congestion gives rise to a change in color, the iris losing its brilliancy and taking on a clouded, dull, lack-lustre appearance, and assumes the different tints of gray, green or red. This alteration in color is due partly to congestion and partly to effusion into its substance.

This effusion is either serum, plastic lymph or pus, and accordingly iritis has been classified into the serous, plastic and purulent.

The effusion is poured into the substance and on the surface of the iris, and passing thence into the aqueous humor renders this more or less turbid. The turbidity of the aqueous is still further increased by the exudation of lymphoid cells and exfoliation of the epithelium of the iris.

These exudations into the aqueous may remain suspended, obscuring vision, or may precipitate to the bottom of the anterior chamber, forming hypopion.

The congestion and effusion into the iris give rise to swelling; this swelling causes it to impinge on the pupil, the pupil thereby becoming smaller.

When the pupil is small, the inner margin of the iris rests on the anterior surface of the lens, and if plastic effusion take place just at this point it glues the inner edge of the iris to the lens.

Thus are formed those adhesions (synechiæ) which are so difficult to break up and which give rise to such tedious relapses.

If a midriatic be now applied the pupil will not dilate smoothly, but will appear notched, the points of adhesion remaining stationary and all the rest dilating, giving the pupil somewhat of a looped appearance.

The points of adhesion vary in number from one to many, and may even entirely encircle the pupil, binding the whole of the inner edge of the iris to the lens, and so cutting off all communication between the anterior and posterior chambers. This condition by preventing drainage from the posterior chamber and vitreous humor, sets up a secondary glaucoma, and may seriously threaten vision.

In severe cases blood-vessels become plainly visible on the front of the iris, and even hemorrhage into the aqueous humor is occasionally observed.

The ocular conjunctiva is considerably injected, and in severe cases occasionally chemotic. The sub-conjunctival vessels are markedly congested, having the appearance of a pink band or zone just posterior to the corneo-scleral junction. The redness diminishes from the cornea toward the oculo-palpebral fold.

In serous iritis, the effusion being serum is devoid of plasticity, therefore few or no synechiæ form. The turbid serum being poured into the aqueous humor faster than its drainage can be effected causes an over-distention of the globe, and a glaucomatous state results.

The intra-ocular pressure upon the ciliary nerves causes their partial or complete paralysis, and hence results a semi-dilated pupil.

Iritis usually begins with a feeling of weakness in the eye, the patient complaining of having a "cold in the eye." To these are soon added a free secretion of tears, a rather congested appearance around the cornea, with some dread of light.

The iris will now present a cloudy appearance, having lost its distinct fibrillated structure, and the pupil being decidedly contracted; nor does it dilate when shielded from light. These appearances become more manifest when the diseased eye is compared with the other or sound one.

If atropine be now applied it will be found that a weak solution (gr. i to aq. ℥i) dilates the pupil tardily or not at all; while to a stronger solution (gr. iv or viii to aq. ℥i) it often yields only after repeated applications. Indeed, the contracted, irregular and sluggish pupil is a most characteristic sign of iritis. This sluggishness of the pupil is due, not only to hyperæmia of the iritic tissue but also to effusion into the substance of the iris.

Pain is usually present, but in varying degrees. It is confined generally to the orbit, temples and forehead, occasionally reaching to the nose and check. It is aggravated during the evening and night, preventing rest and sleep. This pain it would seem is partially due to pressure upon the ciliary nerves, inasmuch as paracentesis often relieves it for the time.

The patient shuns the light, and complains of pain when it is exposed for examination. The tears are described as hot, scalding, and often excoriate the lower lid and inflame the interior of the corresponding side of the nose.

The eye will now present a red, conjested appearance, the redness being confined more to the ball and less to the lid. Upon closer examination the engorged vessels are found beneath the ocular conjunctiva, as is proven by the fact that they remain stationary and allow the conjunctiva to be moved over them.

The cornea may now present a slight cloudiness, due to deposits on its posterior surface taking place from the aqueous humor. There may be more or less fever, with loss of appetite and coated tongue.

In serous iritis the disease is developed less rapidly, often appearing from the first in a subacute or chronic form. It attracts less attention being accompanied by less evident signs of inflammation. The patient is led to seek advice, not for pain, but for loss of vision. The pupil is now semi-dilated and almost immovable, aqueous turbid, and intra-ocular tension increased. There is also a punctated deposit on the lower part of the posterior surface of the cornea.

Iritis may terminate in from one to six weeks, or then becoming chronic may last indefinitely. It is usually confined to one eye, though occasionally, as when due to syphilis, one eye may become affected soon after the other.

The serous form is more chronic and intractable, often requiring months for its relief.

Iritis, as when traumatic, may be simply a local inflammation, confined to the tissue of the iris without the involvement of any other parts of the system. Or it may be the local expression of a constitutional disease, as when due to syphilis, rheumatism, etc.

The causes of iritis may be divided into the local and constitutional.

Among the local causes may be mentioned wounds of whatever kind. Not only wounds of the iris, but of the cornea, may cause it.

Iritis may be caused by extension of inflammation from other tissues, as from the cornea or ciliary bodies; also sympathetically, as in sympathetic ophthalmia, and from deep ulcers or abscesses of the cornea. It is often attributable to a sudden cooling of the body, as in "taking cold," and sometimes to detachment of the retina.

Naming the constitutional causes in the order of their frequency, first comes syphilis, next rheumatism, then scrofula, gonorrhœa and gout.

The relapses of iritis seem to be due largely to the recurrence of the cause of the original inflammation, but still more largely to the irritation kept up by synechiæ formed at some previous attack.

Iritis, if well developed and the cornea clear, is not difficult of diagnosis. The patient complains of pain, dread of light and a watering of the eye.

Upon examination, the discharge is found to consist not of pus but of tears. The circum-corneal zone is injected, the inner side of the lids but slightly reddened. The iris is discolored, the pupil contracted, notched and irregular. On using atropine it dilates slowly, the synechiæ often becoming now plainly marked, or tearing loose leave points of iritic pigment on the lens.

In serous iritis the pupil is semi-dilated and almost immovable. These are less evident signs of inflammation than in the plastic variety. The aqueous is turbid, deposits often having formed on the posterior surface of the cornea, rendering it punctated. The intra-ocular tension is increased and vision correspondingly compromised.

The prognosis will depend largely upon whether, at the time of our first visit, adhesions have formed, between the iris and lens, upon the cause of the disease, and upon the care which the patient can take of himself.

Iritis of mild type, due as for example to cold, seen before adhesions have formed, may be cured in from one to two weeks. The presence of adhesions protract the case, and constitutional causes such as syphilis often retard convalescence.

In a large per cent. of cases the inflammation gets well in from four to six weeks, leaving, perhaps, one or more points of adhesion to the lens, which forever afterwards predispose to a relapse of the iritis.

In the treatment of most inflammations, the first indication is the removal of the cause; in iritis it is different, it is the dilatation of the pupil. This dilatation can usually be effected by putting into the conjunctival sack a one or two per cent. solution of Sulphate of Atropia.

Of this solution one drop should be put in the conjunctival sack every ten minutes until the pupil is dilated, or until eight or ten drops have been instilled. Should the pupil remain contracted, the atropine should be suspended for a couple of hours. During this time, if there is much injection, four or six leeches may be applied to the temple and free after bleeding encouraged. The absorption of the atropia, having been at first prevented by the intense injection and congestion, will now be more readily effected, and cause speedy dilatation.

Failing in this, we might use a four to eight per cent. solution of cocaine. Or, failing in this, we can puncture the cornea, allowing the aqueous to slowly escape, and again apply a two or three per cent. solution of atropine.

By some or all of these measures, the pupil will have dilated more or less, showing likely one or more points of attachment to the lens.

The pupil being dilated, the iris is removed from contact with the lense and hence adhesions between them are prevented. This dilatation should be maintained throughout the disease by the use of atropine. Atropine in iritis has a many-sided beneficial effect: First, it dilates the pupil, breaking up old synechiæ and preventing the formation of new ones; second, it paralyzes that ever active muscle, the sphincter pupillæ, thus allowing the iris to obtain that quiet so necessary to the successful treatment of inflammation; third, it paralyzes the ciliary muscle, thus cutting off one of the predisposing causes of cyclitis; fourth, it relieves pain, allowing rest and sleep; it relieves the dread of light. Should atropine cause irritation, duboisine or hyasciamine may be used instead.

Leeches to the temples, applied two to three times a week, during the acute stage, afford marked relief. Later, blisters alternately to the temple and mastoid are serviceable.

During the day pains may be relieved by bathing the eye in a solution of one drachm of powdered extract belladonna in a pint of hot water. At night opium is often necessary to procure rest and sleep.

Should the pain remain severe, paracentesis of the cornea, allowing the aqueous to escape and relieve pressure, will give temporary relief, and may be repeated if necessary. Should the case remain aggravated iridectomy may be done.

When iritis is caused or accompanied by constitutional diseases, such as syphilis, rheumatism, etc., appropriate constitutional remedies should be at once and vigorously given.

Indeed mercury is often freely given to dissolve the synechiæ and iodide of potassium is considered valuable not only in syphilitic iritis, but in iritis due to various causes.

